# The effect of similarity perceptions on human cooperation and confrontation

**DOI:** 10.1038/s41598-023-46609-8

**Published:** 2023-11-13

**Authors:** Ilan Fischer, Lior Savranevski

**Affiliations:** School of Psychological Sciences, University of Haifa, Haifa, Israel

**Keywords:** Evolution, Psychology

## Abstract

By assuring aversive actions are followed by similarly aversive reactions, legislators of antiquity aimed to reduce belligerence and aggression. In the present study we show how similarity perceptions drive cooperation and confrontation across several strategic decision types. Examining the choices made in three one-shot symmetric conflict games: the prisoner’s dilemma, the chicken, and the battle of the sexes, we show how a short encounter with a stranger accounts for the formation of subjective similarity perceptions, which together with the expected payoffs of the game determine the choice of the preferred alternative. We describe the role of similarity perceptions for all two-by-two games, specifically for a subset of fifty-seven games that are sensitive to similarity perceptions with the opponent. We then suggest that this mechanism, by which individuals maximize expected payoffs, is key to the understanding of the evolution of cooperation and confrontation.

## Introduction

**Figure 1:**
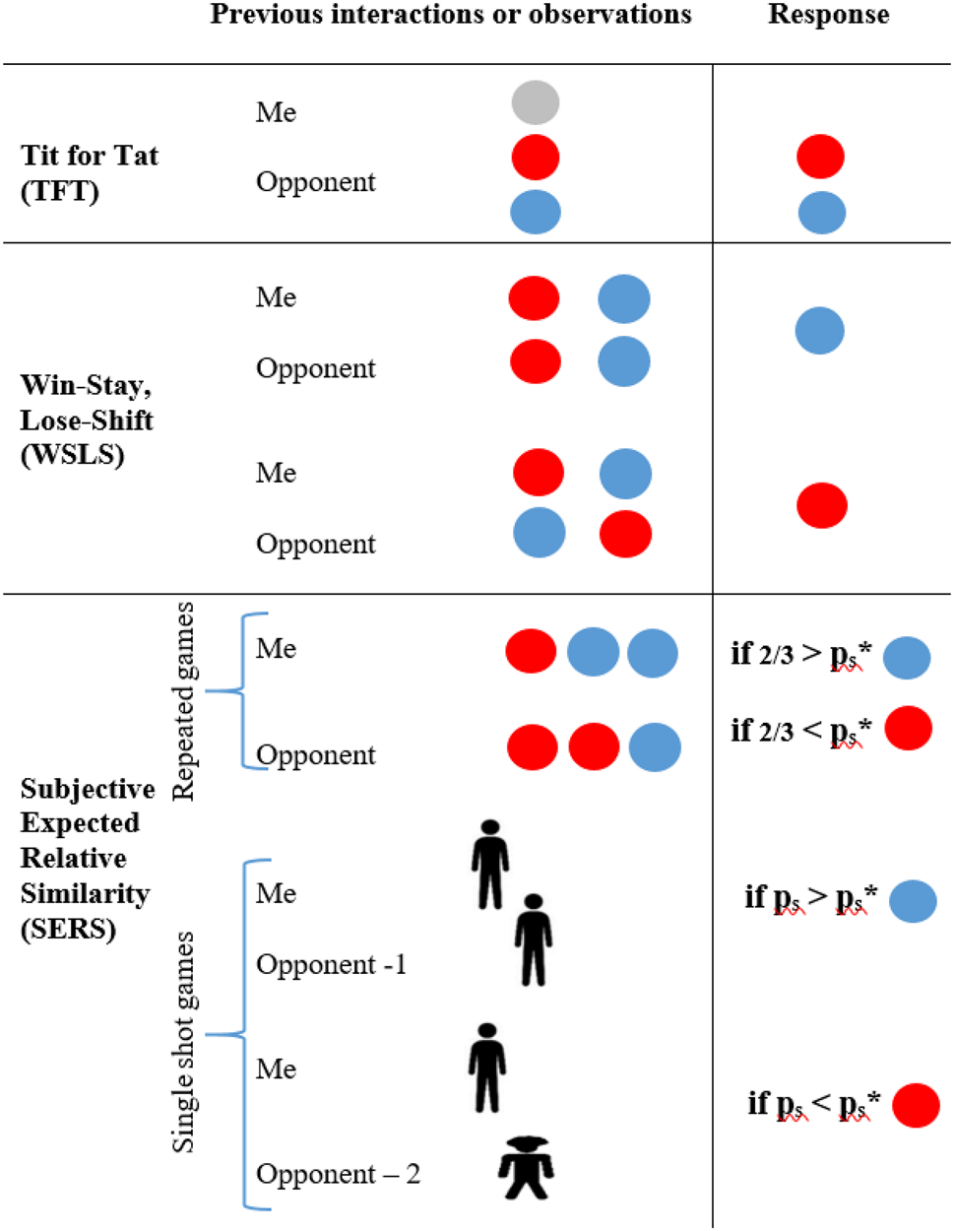
Similarity based strategies. Blue and red circles represent the choice of cooperation and defection, respectively. Grey circles indicate choices that are not relevant for the illustrated strategy. From top to bottom**:** Tit For Tat—mirroring of opponent’s previous choice, either cooperation or defection (following an initial cooperative move). Win Stay, Lose Shift—similar choices, either mutual cooperation or mutual defection, are followed by the choice of cooperation, while different choices are followed by the choice of defection. Subjective Expected Relative Similarity (SERS) for repeated interactions—the extent of strategic similarity, p_s_, is computed from previous interactions and is then compared with the similarity threshold, p_s_*, computed from the game’s payoffs [i.e., for the PD game p_s_* = (t−s)/(t−s + r−p)]. Cooperation is preferred whenever p_s_ > p_s_*. In the repeated game example, two of three previous choices (mutual defection, non-coordinated choices, and mutual cooperation) were identical, therefore cooperation provides a higher expected payoff for the next game if 2/3 > p_s_* and vice versa. Further developing SERS for repeated games, by accounting not only for observed similarity but also for the tendency of the opponent to become similar results in the Mimicry and Relative Similarity strategy^12^. Finally, SERS for single shot games is approximated by individuals using various similarity indicating cues (here illustrated by depictions of similar or different silhouettes) to determine whether the opponent is sufficiently similar.

The principle of action-reaction similarity has been practised throughout human history as a deterrent of aggression. It has been manifested by laws of retributive justice (*lex talionis*;^1^, Fig. 1), where offenses are followed by identical punishments, inflicted by the central authority. Retributive justice was practiced in Mesopotamia, the Arabian Peninsula and the Mediterranean region. It was embedded in the laws of Eshnunna (2000 BCE), the code of Hammurabi (1750 BCE), the Bible (Exodus 21:23–27) and the Quran (Surah 2, 178). But action-reaction similarity may also be generated by the parties themselves, without the intervention of a central authority. If the parties can sustain occasional defeats, then by consistently following the rules of *an eye for an eye* together with *a goodwill for goodwill*, they generate strategic similarity, deter aggressors and promote benevolent interactions. Moreover, the nature and intensity of reciprocated actions also play a critical role in determining the trajectory of future interactions^2^.

Moving from ancient legislation to theoretical strategic models clarifies the efficacy of the action-reaction principle. This has been demonstrated by the well-known Tit for Tat (TFT) strategy that, following initial cooperation, mimics opponents’ choices^3^ (Fig. 1). TFT was shown to outperform many rival strategies in a tournament of repeated prisoner’s dilemma (PD) games^3–6^. Unlike TFT, the Win-Stay Lose-Shift strategy (WSLS, Fig. 1)^7^ does not *generate* similarity, but responds to previously *observed* similarity with the opponent. WSLS responds to identical choices, either mutual cooperation or mutual defection, by choosing to cooperate, and to dissimilar choices by choosing to defect. (The choices of the WSLS strategy are not defined by the authors as responses to similarity^7^. Instead, the reward and the temptation payoffs are regarded as satisfactory outcomes that motivate the repetition of the previous choice (i.e., stay), while the punishment and sucker payoffs are regarded as non-satisfactory outcomes that motivate the change of the previous choice (i.e., switch). The same principle is described by Rapoport and Chammah (1965)^5^ as simple reactions to reinforcement or an aversion to negative payoffs.) While repeated interactions provide clear and observable indications of strategic similarity, many interactions involve only a single forthcoming encounter (or are perceived as such by parties that do not consider the possibility of future encounters). In such single-shot interactions, strategic similarity needs to be inferred from observed or perceived similarity cues that are available at the time of the interaction. After similarity perceptions have been formed, one needs to decide whether their extent is sufficiently high to warrant cooperation, or whether a confrontational choice is likely to provide higher payoffs. To resolve this dilemma, the strategy of Subjective Expected Relative Similarity (SERS) relies on two similarity-related indices (1) the observed or perceived extent of *strategic similarity* with the opponent, expressed by the probability indicating the prospects of both players to choose identical alternatives, denoted by p_s_, and (2) the *similarity threshold* of the interaction, denoted by p_s_*, which is computed from the game’s payoffs and determines the optimal switching point between alternatives^8,9^. SERS prescribes choosing a cooperative alternative whenever the subjective perception of strategic similarity with the opponent, p_s_, exceeds the critical similarity threshold, p_s_*, of the game, otherwise it prescribes choosing the less cooperative (i.e., confrontational) alternative. Since the choice of an alternative is dependent not only on the payoffs, but also on the extent of perceived strategic similarity, an opponent may be regarded as sufficiently similar while playing a specific game, but not sufficiently similar while playing another game with a higher similarity threshold. In a parallel manner, two opponents that evoke different similarity perceptions, may motivate different choices, even when the interaction is modelled by the same game.

As abovementioned, similarity in *single-shot* games cannot be inferred from previous strategic choices that were made along past encounters. Instead, various indirect cues, such as: observed behaviours, attitudes and preferences, reasoning and cognition, derived from both verbal and non-verbal communication, may serve as proxies that help assessing the extent of strategic similarity—the prospects of both parties choosing identical alternatives in the forthcoming interaction. Previous studies have shown that cues related to similar personality traits, matching choices^8,9^, the sense of belonging to a shared nationality^10^, or the alignment with identical person-descriptive words^11^, all have the capacity to foster cooperation in subsequent single-shot games. SERS has provided the logic for playing repeated games, as formalized by the Mimicry and Relative Similarity (MaRS) strategy, which has demonstrated superior performance compared to various prominent strategies and learning algorithms^12^. It was also applied to explain COVID-19 vaccination hesitancy as well as the public’s reaction to the challenges imposed by global warming^13^. In this study, we extend the behavioural scope of SERS by testing whether the reliance on similarity with the opponent and the payoffs of the encounter, as previously observed in PD playing participants^8,9^, represents a more universal decision-making principle.

Considering the taxonomy of two-by-two completely rank-ordered games proposed by Rapoport and Guyer^14^, the PD game is one of 78 different games (representing an extended set of 576 strictly ordered two-by-two games). While *theoretical analyses* show that SERS provides a payoff maximizing decision rule for all two-by-two games, it may recommend making different choices when holding different similarity perceptions of the opponent only for 57 games of the taxonomy, termed Similarity Sensitive Games (SSGs) [^9,13^, Supplementary materials]. To easily identify all 57 SSGs we may compare the choices made under the two boundaries of opponents’ complete strategic similarity, p_s_ = 1 (i.e., both players are assured to choose identical alternatives), and opponents’ complete strategic difference, p_s_ = 0 (i.e., both players are assured to choose different alternatives). If the preferred choice under the extreme assumption of complete similarity differs from the preferred choice under the opposite assumption of complete difference, the game is an SSG. If the preferred choice under the assumption of complete similarity is identical to the preferred choice under the assumption of complete difference, the game is a non-SSG. This simple test shows that 21 games are similarity-sensitive for both row and column players, while 36 games are similarity-sensitive for only one of the players (i.e., the probability of similarity, p_s_, affects the choices of either the row or the column player, but not both) [^9,13^, Supplementary materials]. The set of 57 SSGs comprise six games that are characterized by symmetric payoffs for both players. They comprise three *conflict* games and three *no-conflict* games. (The three *no-conflict* games are characterized by an efficient Pareto equilibrium, a cell where both players jointly obtain their maximal payoff, thus strongly motivating mutual cooperation. Therefore, the impact of SERS on players of the three *no-conflict* games depends on the extent to which participants perceive these games as strategic interactions with conflicting interests of the parties.) The three *conflict* games include the prisoner’s dilemma, the chicken^15^, and a coordination game known as the battle of the sexes^16^. Since similarity has been shown to predict choices in the PD game^8,9^ and since raising similarity perceptions has been proposed as a means for increasing cooperation in both the PD and the chicken game^13^ and since similarity has been shown to increase coordination in matching environments like the battle of the sexes^11^, we expect similarity perceptions of the opponents, to drive behavioural choices in all three *conflict* games, played as single-shot games. More specifically, we expect participants’ choices to be associated with: (1) *naturally arising similarity perceptions*, and (2) *similarity thresholds computed from the payoffs of each game*. Since we are unaware of an existing formal model that merges attitudinal, behavioural and cognitive aspect into a comprehensive measure of perceived similarity, we are unable to calculate objective similarity perceptions for each participant. Instead, we provide participants pairs with the opportunity to perceive various similarity attributes of each other, and then use participants own subjective perceptions to predict their choices. These perceptions may be regarded as individual beliefs, apprehended by the mind and held to be reality^17^ and are hence expected to influence individuals’ choices in the tested games.

We proceed by: (1) describing the PD game as an example for the application of SERS driven payoff-maximizing decisions, (2) formalizing SERS’s generalized structure and its theoretical capacity to provide a payoff-maximizing strategy for other SSGs, and (3) empirically testing SERS’s predictions for eight examples of the three conflict games.

The Prisoner’s Dilemma (PD) game and its SERS prescribed choices.

The PD game^4^ (Table 1) has become a primary model for the study of war, conflict and cooperation^3,5^. It is described by a two-by-two payoff matrix that allows each player to choose either a cooperative or a competitive (defective) alternative. If both players cooperate, each player obtains the Reward (R) payoff. If both defect, each player obtains the Punishment (P) payoff. However, if one of the players defects while the other cooperates, the defector obtains the Temptation (T) payoff and the cooperating player obtains the Sucker (S) payoff, where T > R > P > S (and 2R > T + S, assuring cooperation is constrained to the actions of the players within the game). Defection is regarded as the preferred choice from the perspective of many decision rules. It is both the Maxi-Min and the Maxi-Max solution^16^ of the game. It is also a dominant strategy that allows players to protect themselves from exploitation and still retain the option to exploit a trusting opponent. Moreover, the choice of mutual defection is the only Nash equilibrium of the game—a cell that none of the players is motivated to abandon unilaterally^18,19^; Table 1). Nevertheless, empirical studies have shown that players do not necessarily choose to defect; some choose to cooperate, while others choose to defect^5,20–22^, thus casting doubt on the capacity of theoretical models to predict actual behaviour. Several indices have been proposed in order to explain the extent of cooperation and confrontation. Among others research has focused on: the motivation to cooperate^15,23^, the extent of the conflict of interests among the players^24^, the role of expected average payoffs^25^, the risk embedded in the game^26^, or both risk-averting and gamble-intending characteristics of the payoffs, together generating an index of the dilemma strength^27^.

**Table 1:** Three symmetric conflict games and their respective decision properties.

Type of game	Rank-ordered payoffs		Dominance	Maxi-min	Mini-max Regret	Maxi- max	Lap lace	Nash	Pareto	SERS’s expected values for the row player	SERS’s similarity threshold boundaries
Prisoner's Dilemma	3, 3	1, 4	BB	BB	BB	BB	BB	BB	AA AB BA	p_s_ × 3 + (1−p_s_) × 1 p_s_ × 2 + (1−p_s_) × 4	0.5 < p_s_* < 1
	4, 1	2, 2									
Chicken	3, 3	2, 4	None	AA	AA	BB	AA	AB BA	BA AB AA	p_s_ × 3 + (1−p_s_) × 2 p_s_ × 1 + (1−p_s_) × 4	0 < p_s_* < 1
	4, 2	1, 1									
Battle of the Sexes	2, 2	3, 4	None	AA	AA	BB	AA	BA AB	BA AB	p_s_ × 2 + (1−p_s_) × 3 p_s_ × 1 + (1−p_s_) × 4	0 < p_s_* < 1
	4, 3	1, 1									

The rank ordered payoffs, describe four cells, each showing the payoff of the row player (left) and the column player (right), as derived from the choice of the upper or the lower row and the left or the right column. The notations AA, AB, BA, and BB indicate the upper-left, upper-right, lower-left, and lower-right cells of the game. Each decision property points to one, two or three cells in the matrix, which may be regarded as plausible behavioural predictions. SERS’s expected values are computed while assuming the rank-orders, 1 to 4, represent actual payoffs. The right column shows the lower and upper boundaries of the similarity thresholds, p_s_*, that serve as switching points for SERS’s decision rule (i.e., EV_cooperation_ = EV_defection_).

While these and other critical indices focus on the games payoffs, SERS explains and predicts behavioural choices by taking into account both the payoff values of the game, and the extent of subjectively perceived *strategic similarity* with the opponent—the prospects of both parties choosing identical alternatives in the forthcoming interaction. Using a subjective estimate of strategic similarity, denoted by p_s_, each player may compute the expected payoff for the choices of cooperation and defection. The expected value of cooperation is given by EV_cooperation_ = $R \times p_{s} + S \times (1 - p_{s} )$, and the expected value of defection is given by EV_defection_ = $P \times p_{s} + T \times (1 - p_{s} )$. Comparing EV_cooperation_ with EV_defection_ allows choosing the alternative that provides a higher expected value. Hence, cooperation should be preferred whenever $R \times p_{s} + S \times (1 - p_{s} ) > P \times p_{s} + T \times (1 - p_{s} )$, and defection should be preferred whenever $R \times p_{s} + S \times (1 - p_{s} ) < P \times p_{s} + T \times (1 - p_{s} )$. These decision rules may also be expressed as follows: cooperate if $p_{s} > \frac{T - S}{{T - S + R - P}}$, be indifferent if $p_{s} = \frac{T - S}{{T - S + R - P}}$, otherwise defect. Since the ratio $\frac{T - S}{{T - S + R - P}}$ is derived from the game’s payoff matrix, it provides an *objective* criterion for comparison with the estimated subjective probability of strategic similarity. Thus, defining the critical similarity threshold of the game as $p_{s} * = \frac{T - S}{{T - S + R - P}}$, we obtain a simple decision rule: cooperate if $p_{s} > p_{s} *$, be indifferent if $p_{s} = p_{s} *$, otherwise defect.

Note that the computation of EVs in SERS differs from regular expected values. While the calculation of regular EVs relies on the prospects associated with a specific choice of the opponent, such as the prospects of the opponent to cooperate or to defect in the PD game, SERS computes EVs by relying on the *probability of strategic similarity*, p_s_, which refers to the prospects of the parties making identical choices (i.e., both choosing to cooperate or both choosing to defect in the PD game).

### Generalizing SERS

In the present study we generalize SERS by addressing symmetric games that provide each player with the same strategic problem^28^. We define row and column players’ alternatives as A and B. Hence, when both choose A or both choose B (i.e., AA or BB), both choose identical alternatives, which are reflected in identical payoffs (while the choice of AB and BA indicates the choice of different alternatives). Note that each *symmetric* two-by-two game matrix has four possible permutations generated by switching rows, columns or both rows and columns. These permutations require adjusting SERS’s computation by realigning payoffs with players corresponding perceptions of strategic similarity, p_s_ (and the complementary 1−p_s_ values). Hereafter, we present all games and corresponding computations in accordance with their generic presentation in Rapoport and Guyer’s taxonomy^14^.

**Figure 2:**
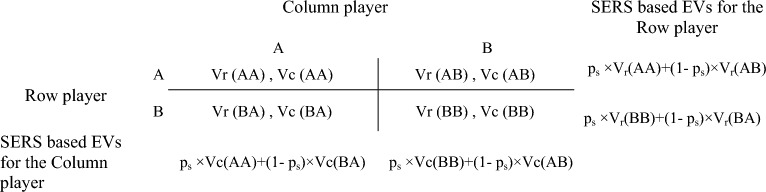
A symmetric two-by-two single-shot game depicting the payoff values for the row (V_r_) and the column player (V_c_) for each of the four choice combinations. SERS based EVs are computed from the payoff values, the subjectively perceived strategic similarity with the opponent, p_s_, and the complementary extent of perceived strategic difference, 1−p_s_.

The payoff values assigned to the row, V_r_, and column, V_c_, players for each of the four matrix cells are denoted by: V_r_ (A,A), V_r_ (A,B), V_r_ (B, A), V_r_ (B,B) for the row player; and by V_c_ (A,A), V_c_ (A,B), V_c_ (B, A), V_c_ (B,B) for the column player. SERS’s decision rule, from the perspective of the row player (where V refers to V_r_), is hence defined as follows:

Choose A if $$p_{s} \times V(AA) + (1 - p_{s} ) \times V(AB) > p_{s} \times V(BB) + (1 - p_{s} ) \times V(BA)$$,

be indifferent if $p_{s} \times V(AA) + (1 - p_{s} ) \times V(AB) = p_{s} \times V(BB) + (1 - p_{s} ) \times V(BA)$, otherwise choose B. Further defining p_s_* = $\frac{V(BA) - V(AB)}{{V(BA) - V(AB) + V(AA) - V(BB)}}$ generates the abridged decision rule: choose A if p_s_ > p_s_ *, be indifferent if p_s_ = p_s_*, otherwise choose B. Figure 2 depicts a symmetric two-by-two game (i.e., V_r_(AA) = V_c_(AA), V_r_(BB) = V_c_(BB), V_r_(AB) = V_c_(BA), and V_r_(BA) = V_c_(AB)). (Note that in symmetric games such as the described PD game similar choices are clearly defined (i.e., the AA and BB cells in the standard representation of the game), but when applying SERS to non-symmetric games, one has first to identify similar cells before associating them with the perceived similarity index, p_s_. The identification of more and less similar cells may rely on the proximity of the payoffs in these cells.)

Clearly, SERS’s predictions are not always different from the predictions derived from other decision rules. Considering the inherent uncertainty associated with the expected outcomes of games, the relationship between risk analysis and game theory could be deemed complementary or mutually reinforcing^29^. Furthermore, empirical research has revealed a correlation between individuals' risk perception and the inherent risk embedded within the game's payoffs, indicating that individuals with a higher tolerance for risk are more inclined to cooperate in games with greater risk factors. Conversely, those leaning toward risk aversion tend to prefer cooperation in low-risk games^30^. Thus, we compare the predictions of SERS with decision rules that encompass three fundamental risk attitudes. We examine risk aversion as represented by the maxi-min and the mini-max regret principles^19,31^, risk seeking as represented by the maxi-max^19^, and risk neutrality as represented by Laplace’s principle of insufficient reason^32^. We also examine fundamental rationality as portrayed by the dominance principle, and also point to the existence of Nash^18^ and Pareto equilibria outcomes^14^. Table 1 shows the predictions derived from seven fundamental decision properties and principles for each of the three SSGs tested in the present study. These include the following: (1) the Dominance property, where the player chooses the alternative that provides a better payoff under both possible choices of the opponent (if such a dominant alternative exists); (2) the Maximin principle, where the player identifies the worst possible payoffs that might be obtained while selecting each of the alternatives, and then chooses the alternative that contains the maximum of both minima^19^; (3) the Minimax Regret principle, where the payoff matrix is transformed into a matrix of regret values (i.e., each value represents the loss relative to the best outcome under the specific choice of the opponent), associating each alternative with its *maximally possible regret* value. This allows the player to choose the alternative that provides the minimum of both *maximal regret* values^31^; (4) the Maximax principle, where the player may identify the best possible payoffs obtained while selecting each alternative, and chooses the alternative that contains the maximum of both maxima^19^; (5) Laplace’s principle of insufficient reason, where the player ignores the strategic nature of the game by assigning equal probabilities to each choice of the opponent and choosing the alternative that provides the highest expected value^32^; (6) the Nash equilibrium, which is a property of a cell where none of the players is motivated to change his or her chosen alternative (i.e., assuming the other player does not deviate from his or her choice;^18^); (7) the Pareto equilibrium, also a property of a cell in which both players’ payoffs cannot be *jointly* improved by moving to another cell (i.e., there is no other cell in the game where *both* players obtain higher payoffs^14^); (8) since SERS requires also knowing the values of *subjective* perceptions of similarity with the opponent, Table 1 shows the two SERS-based expected values and the theoretical boundaries of the similarity thresholds, p_s_*, for each game. As shown in Table 1, the most consistent choice across all mentioned decision rules, apart from SERS, is the choice of defection for the PD game. Nevertheless, the common prediction of *defection* (lower row and right column) for the PD game is inconsistent with empirical observations that reveal many instances of cooperative choices^8,9,20,33^.

Notably, Table 1 does not exhaust all possible decision rules, specifically not rules that involve more complex reasoning. For example, Stahl and Wilson^34^ proposed a theory of bounded rational strategic thinking in which players are distinguished by their model of other players and their ability to identify optimal choices given their priors. Mengel showed how indices of risk and temptation explain variation in cooperation rates^33^. Liberman, Samuels and Ross tested the effect of game labelling^35^, and Bornstein incorporated both intra- and intergroup conflict motivations^21^.

Here we predict that participants of all three symmetric single-shot conflict games (i) cooperate more often while interacting in games with lower *similarity thresholds,* and (ii) cooperate more often while *subjectively perceiving* a higher extent of similarity with the opponent. Importantly, by presenting participants with different exemplars of each game we exert experimental control on the values of similarity thresholds. However, we do neither manipulate or induce similarity perceptions. Instead, we associate subjective similarity perceptions of the participants with their corresponding strategic choices.

## Method

To test the associations of similarity thresholds, p_s_*, with cooperation rates across several examples of the three games, we first transformed the rank-ordered values (i.e., 1–4, Table 2) in each of the cells into several sets of continuous payoffs. We generated three examples of the chicken and three exemplars of the battle of the sexes game, each defined by its corresponding p_s_* index (p_s_* = 0.35, 0.61 and 0.83). Since the PD game is constrained by a minimum of p_s_* = 0.5,  it is represented by two exemplars (p_s_* = 0.61 and 0.83), generating a total of eight games (Table 2) (Note that, the PD game is defined by the inequalities of T > R > P > S, and its p_s_* = (T−S)/(T−S + R−P). Allowing R → T, and P → S, we obtain p_s_* → (T−S) /2(T−S) = 0.5. Alternatively, allowing R → P, we obtain p_s_* → (T−S)/(T−S) = 1. Hence, p_s_* values for PD games are constrained by the inequalities of 0.5 < p_s_* < 1, while p_s_* values for the chicken and the BoS games are constrained by the inequalities of 0 < p_s_* < 1.) To keep the payoff values within the same range, all eight games were given identical minima (v = 1) and maxima payoff values (v = 20). These eight games served as stimuli for the study, allowing to test both the impact of objective similarity thresholds and subjective similarity perceptions. All participants played only a single game with their partner.

**Table 2:** Rank ordered formulations, continuous payoffs and their respective similarity thresholds, for eight games tested in the study.

Similarity Sensitive Games (SSG)	Ordinal payoffs		Low threshold games		Mid threshold games		High threshold games		p_s_* theoretical boundaries
Prisoner’s Dilemma	3, 3	1, 4			16, 16	1, 20	16, 16	1, 20	0.5 < p_s_* < 1
	4, 1	2, 2			20, 1	4, 4	20, 1	12, 12	
					p_s_ * 0.61		p_s_ * = 0.83		
Chicken	3, 3	2, 4	16, 16	12, 20	11, 11	4, 20	5, 5	2, 20	0 < p_s_* < 1
	4, 2	1, 1	20, 12	1, 1	20, 4	1, 1	20, 2	1, 1	
			p_s_* = 0.35		p_s_ * = 0.61		p_s_ * = 0.82		
Battle of the Sexes	2, 2	3, 4	12, 12	14, 20	6, 6	12, 20	3, 3	10, 20	0 < p_s_* < 1
	4, 3	1, 1	20, 14	1, 1	20, 12	1, 1	20, 10	1, 1	
			p_s_ * = 0.35		p_s_ * = 0.61		p_s_ * = 0.83		

Similarity thresholds, p_s_*, calculation for the PD games: (20–1)/(20–1 + 16–4) = 0.61, and (20–1)/(20–1 + 16–12) = 0.83; for the chicken games (20–12)/(20–12 + 16–1) = 0.35, (20–4)/(20–4 + 11–1) = 0.61, and (20–2)/(20–2 + 5–1) = 0.81; for the battle of the sexes games (20–14)/(20–14 + 12–1) = 0.35, (20–12)/(20–12 + 6–1) = 0.61, and (20–10)/(20–10 + 3–1) = 0.83.

Five hundred and four participants studying at various university departments (average age 23.78, 29% males) were recruited by the university’s online recruitment system, and randomly-assigned to one of eight games. They were rewarded by a show-up fee, supplemented by performance-contingent payoffs that were derived from the game’s outcome. Before interacting in a single-shot game, participant pairs were invited to the laboratory and asked to take part in a guessing assignment, providing the opportunity to observe each other’s behaviour and to form subjective, natural and non-manipulated perceptions of similarity. After being introduced to the study and signing informed consent forms, participants were seated side by side in front of a table separated by a small curtain. They were shown twelve series, each comprising a sequence of # and @ symbols with various proportions. Then, both participants were asked to individually guess the most plausible source that may have generated each sequence, either a sequence of *baskets and misses* made by a basketball player or a sequence of *heads and tails* generated by a repeatedly tossed coin^36,37^. To indicate their response, each participant chose either a card showing a basketball or a card showing a coin. To enable a separate focus on behaviours and cognitions, the choices made along the first six trials remained concealed, thus allowing the participants to observe only their partner’s conduct. Then, for the next six trials, participants handed over the cards with their selected responses to the experimenter, who attached them to a clearly visible board, thus exposing the similarities and differences emanating from participants’ reasoning and providing cues of cognitive similarity. Next, participants were introduced to an example of a two-by-two matrix game. Following an explanation and a comprehension test that assured participants understood the game and correctly identified the eight payoffs, the pairs were separated and each participant was seated in an individual cubicle. They were then asked to play a single game (i.e., one of the eight two-by-two games shown in Table 2) by choosing their preferred alternative, writing it down on paper and sealing it in an envelope. After handing over their choice, but before learning about the opponent’s choice and the game’s outcome, each participant assessed the similarity with the opponent by making a mark along a bounded and nonnumeric scale (labelled ‘not similar at all’ and ‘maximally similar’ at its boundaries). To focus participants on the perceptions that are directly relevant to the notion of strategic similarity in SERS, they were asked to estimate the degree to which the thought processes of the other participant resembled their own. Finally, the game’s outcome was announced, and the obtained payoffs, supplemented by a show-up fee, were paid separately to each participant (see supplemental materials).

All methods were carried out in accordance with relevant guidelines and regulation and were approved by the University Ethics committee—Institutional Review Board (UH IRB14912). All participants provided informed consent.

## Results

### Effects of similarity thresholds

As predicted for all three games, the percentage of cooperative choices was inversely associated with the games’ *similarity threshold*, p_s_*. For the PD game, the thresholds of p_s_* = 0.61 and 0.83 resulted in corresponding cooperation percentages of 73.6 and 43.5 percent (χ^2^ (1,140) = 13.21, p < 0.05, exact Fisher’s test p = 0.000). For the chicken game the thresholds of 0.35, 0.61 and 0.82 resulted in corresponding cooperation percentages of 95.2, 62.7, and 54.7 percent (χ^2^ (2,185) = 27.71, p < 0.05, exact Fisher’s test p = 0.000). For the battle of the sexes game the thresholds of 0.35, 0.61 and 0.83 resulted in corresponding cooperation percentages of 80, 47.4, and 41.9 percent (χ^2^ (2,179) = 20.76, p < 0.05, exact Fisher’s test p = 0.000).

**Figure 3:**
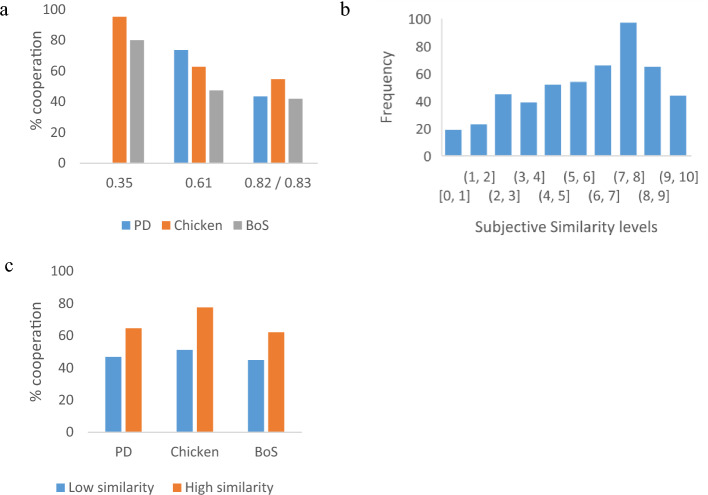
(**a**) The effect of similarity thresholds, p_s_*, on the percent of cooperative choices across three conflict games: the prisoner’s dilemma (PD), chicken, and the battle of the sexes (BoS), (**b**) the distribution of subjective similarity levels across ten categories, each marked by its lower and upper boundary, and (**c**) the effect of similarity perceptions, p_s_, on the percent of cooperative choices across the three conflict games.

Overall, choices across all three conflict games confirmed the SERS-driven prediction, showing an inverse association of similarity thresholds and cooperation rates. The lower the similarity threshold, p_s_*, the higher the percent of cooperative choices (Fig. 3a).

### Effect of subjective similarity perceptions

To test whether similarity ratings of the opponent generated a sufficiently wide distribution of subjective similarity, we classified similarity values into ten categories, covering the range from 0 to 10. Figure 3b shows the distribution of subjective similarity perceptions provided by the participants, revealing a skewed distribution, with an average similarity rating of 6.0 and a median of 6.4. Then to test whether natural similarity perceptions as provided by participants’ ratings serve as indicators of the probability of strategic similarity (i.e., the prospects of both parties making identical decisions) and therefore also as determinants of cooperation and confrontation, we split the scale of reported similarity perceptions into two ranges of low and high similarity (0—49 and 50–100) and compared the frequency of cooperative choices.

This split reflects the underlying assumption of a monotonically increasing association of participants’ behavioral responses and the theoretic concept of strategic similarity (but it does not necessarily imply a linear relation). For the PD game, the low and high similarity perceptions generated cooperation percentages of 46.8 and 64.5 percent (χ^2^ (1) = 4.03, p < 0.05, exact Fisher’s test p = 0.048). For the chicken game, low and high similarity perceptions generated cooperation percentages of 51.1 and 77.5 percent (χ^2^ (1) = 11.89, p < 0.01, exact Fisher’s test p = 0.001). For the Battle of the Sexes game, the low- and high-similarity perceptions generated cooperation percentages of 44.8 and 62.0 (χ^2^ (1) = 4.69, p < 0.05, exact Fisher’s test p = 0.037). Overall, those who reported high similarity perceptions cooperated 21.38 percent more often than those who reported low similarity perceptions (χ^2^ (1) = 20.66, p < 0.01, exact Fisher’s test p < 0.000), thus confirming the second SERS-driven hypothesis across all three conflict games (Fig. 3c).

## Discussion

Looking back through history, similarity seems to have played a meaningful role in moderating human cooperation and confrontation; it has shaped strategic reasoning by promoting an ‘eye for an eye’ and a ‘good will for a good will’ as mechanisms that deter aggression and promote reciprocity. It has also driven the more recent development of strategies such as Tit-for-Tat^3^, Win-Stay Lose-Shift^7^ and Mimicry and Relative Similarity^12^. Clearly, there is no direct link between the antiquity, intuitive human behaviour and purposefully designed algorithms. What links them together is the underlying role of similarity, which allows telling apart friends from foes and motivating either cooperative or confrontational behaviours. As shown by SERS^8,9^ these behaviours depend not only on the extent of similarity, but also on the possible consequences, portrayed by the payoffs associated with each outcome. These two aspects have driven the choices made in three single-shot conflict games tested in the present study, showing that participants’ decisions were determined both by similarity thresholds, p_s_*, and by the participants’ subjective perceptions of similarity, p_s_.

In the present study we have focused on first encounters, or single-shot games. Therefore, participants could not consider choices made in previous encounters, and were driven to rely on various available cues as proxies and indicators of strategic similarity. To this end we allowed participants pairs to observe each other’s appearance, conduct, behaviour and reasoning process, before engaging in a single strategic game. In spite of all participants forming a rather homogeneous sample, their *subjective* similarity perceptions revealed an inverse u-shaped distribution, skewed towards the upper end of the similarity range. Some participants perceived their opponents as being very different from themselves, while others perceived rather high similarity levels. As the only objective indicator of similarity was the observed set of six (out of twelve) responses to the guessing assignment, we may examine its contribution by computing the correlation between the number of identical responses and the reported perceptions of subjective similarities. The correlation of both measures is r = 0.37 (p < 0.01), pointing to a meaningful role of the objective observation, but also suggesting that other indicators were involved in the estimation of opponent’s similarity.

Undoubtedly, the development and empirical testing of a behavioral model that addresses interpersonal and intergroup similarity perception is a worthwhile task. Such research could contribute to the effective design of conflict interventions, particularly in situations where altering payoffs is challenging or unfeasible, leading to relatively fixed similarity thresholds. These interventions could benefit from (1) the selection of negotiators and mediators who are perceived as similar to the opposing party, (2) the deconstruction of disputed issues, with an initial focus on concerns that can be described by payoffs having comparatively low similarity thresholds, potentially allowing for cooperative resolutions based on the perceived level of similarity, and (3) the promotion of activities aimed at fostering similarity among the involved parties before addressing contentious issues.

Unlike the hereby tested single-shot games, many real life interactions span over several repeated encounters, therefore allowing the parties to observe and assess the unfolding extent of strategic similarity (Fig. 1). It seems reasonable to assume that newly formed similarity perceptions will update or override previously formed similarity perceptions. Parties that experience repeated choices of mutual cooperation are likely to raise similarity perceptions of each other and be motivated to cooperate in the future. However, it may seem less intuitive to expect rising similarity perceptions following the experience of mutual defection. To understand the impact of mutual defection one has to distinguish between two cases: (1) laboratory experiments that do not involve meaningful harms or the arousal of intense negative emotions, and (2) authentic disputes, conflicts, and wars that entail significant consequences, negative emotions, and hostile attitudes. In the first case, mutual defection may serve as a clear indicator of strategic similarity and should indeed motivate cooperation. This pattern has been clearly revealed across all repeated games tested by Rapoport and Chamma^5^. The researchers reported observing increasing numbers of *matched responses* as the games progress, most of them comprised from mutual defection, suggesting that paired players become more similar to each other (p. 102). They also reported observing a ‘breakdown’, a switching point where choices of mutual defection begin to decrease, giving way to increasing numbers of mutually cooperative choices (p. 96). The second case of authentic disputes is more complex, since repeated interactions of real conflicts give rise not only to changes in similarity perceptions, but may also change the payoffs structure of the following encounters. If strategic similarity is obtained via mutual defection and both parties have experienced loss of property, territories and lives, a new set of payoffs, shaped not only by raising similarity, but also by negative emotions, vengeance, and spite is likely to emerge. Moreover, if meaningful decisions have already been made, the updating of similarity perceptions may be impaired by conformity and cognitive dissonance that drives individuals to justify previous choices by denying contradictory evidence^38^.

While further empirical testing is required under different payoff matrices, experimental environments, and paradigms, the confirmation of SERS's hypotheses in the present study suggests exploring how these hypotheses might be integrated into (1) the broader game-theoretic framework and (2) the evolutionary perspective of kin and group selection^39^.

This study investigated three prominent and symmetric conflict games. Theoretical analyses and empirical testing could be expanded to encompass all two-by-two games, whether symmetric or non-symmetric, and could potentially be further developed to accommodate n-by-n games as well. Out of the 78 two-by-two rank-ordered games listed in Rapoport and Guyer’s taxonomy [^14^; Supplementary materials] 57 games are similarity-sensitive. These games comprise: (1) the three critical symmetric conflict games tested in the present study; (2) three symmetric no-conflict games, which are characterized by an efficient Pareto equilibrium^14^, (3) 15 non-symmetric conflict games (i.e., games that provide the players with strategic problems that are not fully identical) that are similarity sensitive for both players; (4) 36 games that are similarity sensitive for only one of the parties. Extending SERS to non-symmetric games requires first to identify the cells that represent more similar choices. These cells are then associated with the probability of similarity, p_s_, while the other cells are associated with the complementary probability, 1−p_s_, thus allowing calculating SERS based expected values. The identification of more and less similar cells may rely on the proximity of the payoffs in these cells. Moreover, the remaining 21 non-similarity-sensitive games may also be optimized by SERS, but for these games the payoff-maximizing choice remains the same across all potential perceptions of similarity.

The current study focuses on examining the impact of strategic similarity within simple two-by-two games conducted in laboratory settings. However, the broader evolutionary perspective delves into the emergence and stabilization of cooperative relations among organisms that are genetically or socially related^39^. Defining an uncertain or probabilistic estimate of genetic (or group) relatedness to another individual by the probability p_r_, and denoting a set of four payoffs (a ,b, c, d), associated with the choices of: *cooperation* following correct identification of relatedness (i.e., correct acceptance), c*ooperation* following incorrect identification of relatedness (i.e., false acceptance), *defection* following incorrect identification of non-relatedness (i.e., false rejection), and *defection* following correct identification of non-relatedness (i.e., correct rejection), we may derive a decision rule that recommends cooperation whenever: ap_r_ + b(1−p_r_) > cp_r_ + d (1−p_r_) or whenever p_r_ > (d−b)/(d-b + a−c) ]8]. In other words, similar to SERS, the expected payoffs of the interaction set a crucial benchmark that must be weighed against the probability of relatedness, p_r_. However, genetic or group relatedness in natural environments remains a concealed trait that is not readily discernible. Its assessment is likely to depend on the observation of physical, behavioral and cognitive characteristics, akin to those accessible to the participants in the current study. Therefore, p_s_ may be regarded as an estimate of p_r_, and SERS’s decision rule can be seen as reflecting the same rationale that underlies evolutionary selection models. Consequently, individuals and groups that evolve to make SERS-based decisions are expected to converge on optimal and efficient solutions^40,41^ and maximize their fitness.

To further validate SERS’s hypotheses in ecologically valid settings, future research should develop behavioural procedures for eliciting payoffs and game-structures that reflect the perceptions of the interacting parties, study behavioural models of perceived similarity, and examine the associated decision-making processes.

### Supplementary Information

Supplementary Information.

## Data Availability

All data describing participants’ choices and strategic similarity perceptions across all eight games are available at: https://osf.io/xtjba/?view_only=2d73eb8ed14f43d9a21a7c03dda1937a. List of two-by-two similarity sensitive games and experimental instructions are available in the supplementary materials file.
