# Supplementary material for: The effect of similarity perceptions on human cooperation and confrontation
Source: Sci Rep. 2023 Nov 13;13:19849. doi: 10.1038/s41598-023-46609-8 (PMC10645908; doi:10.1038/s41598-023-46609-8)
Supplement: Supplementary file 1 — Supplementary Information. [file 41598_2023_46609_MOESM1_ESM.docx]

**The effect of similarity perceptions on human cooperation and confrontation**

**Supplementary materials**

**Table S1 – Similarity sensitive game properties**

|  |  |  | **Row and Column Players’ Rank-ordered Payoffs** | | | | |  | **SS for Row** | **SS for Column** |
| --- | --- | --- | --- | --- | --- | --- | --- | --- | --- | --- |
| **Game No.** | **AA row** | **AA col** | **AB row** | **AB col** | **BA row** | **BA col** | **BB row** | **BB col** |  |  |
| **1** | **4** | **4** | **3** | **3** | **2** | **2** | **1** | **1** |  | **٧** |
| **2** | **4** | **4** | **3** | **3** | **1** | **2** | **2** | **1** |  | **٧** |
| **3** | **4** | **4** | **3** | **2** | **2** | **3** | **1** | **1** |  |  |
| **4** | **4** | **4** | **3** | **2** | **1** | **3** | **2** | **1** |  |  |
| **5** | **4** | **4** | **3** | **1** | **1** | **3** | **2** | **2** |  |  |
| **6** | **4** | **4** | **2** | **3** | **3** | **2** | **1** | **1** | **٧** | **٧** |
| **7** | **3** | **3** | **4** | **2** | **2** | **4** | **1** | **1** |  |  |
| **8** | **3** | **3** | **4** | **2** | **1** | **4** | **2** | **1** |  |  |
| **9** | **3** | **3** | **4** | **1** | **1** | **4** | **2** | **2** |  |  |
| **10** | **2** | **3** | **4** | **2** | **1** | **4** | **3** | **1** | **٧** |  |
| **11** | **2** | **3** | **4** | **1** | **1** | **4** | **3** | **2** | **٧** |  |
| **12** | **2** | **2** | **4** | **1** | **1** | **4** | **3** | **3** | **٧** | **٧** |
| **13** | **3** | **4** | **4** | **2** | **2** | **3** | **1** | **1** |  |  |
| **14** | **3** | **4** | **4** | **2** | **1** | **3** | **2** | **1** |  |  |
| **15** | **3** | **4** | **4** | **1** | **2** | **3** | **1** | **2** |  |  |
| **16** | **3** | **4** | **4** | **1** | **1** | **3** | **2** | **2** |  |  |
| **17** | **2** | **4** | **4** | **2** | **1** | **3** | **3** | **1** | **٧** |  |
| **18** | **2** | **4** | **4** | **1** | **1** | **3** | **3** | **2** | **٧** |  |
| **19** | **3** | **4** | **4** | **3** | **1** | **2** | **2** | **1** |  | **٧** |
| **20** | **3** | **4** | **4** | **3** | **2** | **2** | **1** | **1** |  | **٧** |
| **21** | **2** | **4** | **4** | **3** | **1** | **2** | **3** | **1** | **٧** | **٧** |
| **22** | **4** | **4** | **3** | **3** | **2** | **1** | **1** | **2** |  | **٧** |
| **23** | **4** | **4** | **3** | **3** | **1** | **1** | **2** | **2** |  | **٧** |
| **24** | **4** | **4** | **3** | **2** | **2** | **1** | **1** | **3** |  | **٧** |
| **25** | **4** | **4** | **3** | **2** | **1** | **1** | **2** | **3** |  | **٧** |
| **26** | **4** | **4** | **2** | **3** | **3** | **1** | **1** | **2** | **٧** | **٧** |
| **27** | **4** | **4** | **2** | **2** | **3** | **1** | **1** | **3** | **٧** | **٧** |
| **28** | **4** | **4** | **3** | **1** | **2** | **2** | **1** | **3** |  |  |
| **29** | **4** | **4** | **3** | **1** | **1** | **2** | **2** | **3** |  |  |
| **30** | **4** | **4** | **2** | **1** | **3** | **2** | **1** | **3** | **٧** |  |
| **31** | **3** | **4** | **2** | **2** | **1** | **3** | **4** | **1** | **٧** |  |
| **32** | **3** | **4** | **2** | **1** | **1** | **3** | **4** | **2** | **٧** |  |
| **33** | **3** | **4** | **1** | **2** | **2** | **3** | **4** | **1** |  |  |
| **34** | **3** | **4** | **1** | **1** | **2** | **3** | **4** | **2** |  |  |
| **35** | **2** | **4** | **3** | **2** | **1** | **3** | **4** | **1** | **٧** |  |
| **36** | **2** | **4** | **3** | **1** | **1** | **3** | **4** | **2** | **٧** |  |
| **37** | **3** | **4** | **2** | **3** | **1** | **2** | **4** | **1** | **٧** | **٧** |
| **38** | **3** | **4** | **1** | **3** | **2** | **2** | **4** | **1** |  | **٧** |
| **39** | **2** | **4** | **3** | **3** | **1** | **2** | **4** | **1** | **٧** | **٧** |
| **40** | **3** | **4** | **4** | **1** | **2** | **2** | **1** | **3** |  |  |
| **41** | **3** | **4** | **4** | **1** | **1** | **2** | **2** | **3** |  |  |
| **42** | **3** | **3** | **4** | **1** | **2** | **2** | **1** | **4** |  | **٧** |
| **43** | **3** | **3** | **4** | **1** | **1** | **2** | **2** | **4** |  | **٧** |
| **44** | **2** | **4** | **4** | **1** | **1** | **2** | **3** | **3** | **٧** |  |
| **45** | **3** | **2** | **4** | **1** | **2** | **3** | **4** | **1** | **٧** |  |
| **46** | **3** | **2** | **4** | **1** | **1** | **3** | **2** | **4** |  | **٧** |
| **47** | **2** | **3** | **4** | **1** | **1** | **2** | **3** | **4** | **٧** | **٧** |
| **48** | **2** | **2** | **4** | **1** | **1** | **3** | **3** | **4** | **٧** | **٧** |
| **49** | **3** | **4** | **4** | **3** | **2** | **1** | **1** | **2** |  | **٧** |
| **50** | **3** | **4** | **4** | **3** | **1** | **1** | **2** | **2** |  | **٧** |
| **51** | **3** | **4** | **4** | **2** | **2** | **1** | **1** | **3** |  | **٧** |
| **52** | **3** | **4** | **4** | **2** | **1** | **1** | **2** | **3** |  | **٧** |
| **53** | **3** | **3** | **4** | **2** | **2** | **1** | **1** | **4** |  |  |
| **54** | **3** | **3** | **4** | **2** | **1** | **1** | **2** | **4** |  |  |
| **55** | **2** | **4** | **4** | **3** | **1** | **1** | **3** | **2** | **٧** | **٧** |
| **56** | **2** | **4** | **4** | **2** | **1** | **1** | **3** | **3** | **٧** | **٧** |
| **57** | **2** | **3** | **4** | **2** | **1** | **1** | **3** | **4** | **٧** |  |
| **58** | **4** | **4** | **2** | **3** | **1** | **1** | **3** | **2** |  | **٧** |
| **59** | **4** | **4** | **2** | **2** | **1** | **1** | **3** | **3** |  | **٧** |
| **60** | **4** | **4** | **2** | **1** | **1** | **2** | **3** | **3** |  |  |
| **61** | **4** | **4** | **1** | **3** | **3** | **1** | **2** | **2** | **٧** | **٧** |
| **62** | **4** | **4** | **1** | **2** | **3** | **1** | **2** | **3** | **٧** | **٧** |
| **63** | **4** | **4** | **1** | **2** | **2** | **1** | **3** | **3** | **٧** | **٧** |
| **64** | **3** | **4** | **2** | **1** | **1** | **2** | **4** | **3** | **٧** |  |
| **65** | **2** | **4** | **3** | **1** | **1** | **2** | **4** | **3** | **٧** |  |
| **66** | **3** | **3** | **2** | **4** | **4** | **2** | **1** | **1** | **٧** | **٧** |
| **67** | **2** | **3** | **3** | **4** | **4** | **2** | **1** | **1** | **٧** | **٧** |
| **68** | **2** | **2** | **3** | **4** | **4** | **3** | **1** | **1** | **٧** | **٧** |
| **69** | **2** | **2** | **4** | **3** | **3** | **4** | **1** | **1** |  |  |
| **70** | **3** | **4** | **2** | **1** | **4** | **2** | **1** | **3** | **٧** |  |
| **71** | **3** | **3** | **2** | **1** | **4** | **2** | **1** | **4** | **٧** | **٧** |
| **72** | **3** | **2** | **2** | **1** | **4** | **3** | **1** | **4** | **٧** | **٧** |
| **73** | **2** | **4** | **4** | **1** | **3** | **2** | **1** | **3** |  |  |
| **74** | **2** | **4** | **3** | **1** | **4** | **2** | **1** | **3** | **٧** |  |
| **75** | **2** | **3** | **4** | **1** | **3** | **2** | **1** | **4** |  | **٧** |
| **76** | **2** | **3** | **3** | **1** | **4** | **2** | **1** | **4** | **٧** | **٧** |
| **77** | **2** | **2** | **4** | **1** | **3** | **3** | **1** | **4** |  | **٧** |
| **78** | **2** | **2** | **3** | **1** | **4** | **3** | **1** | **4** | **٧** | **٧** |

Rank ordered payoffs for 78 games (representing a wider set of 576 games) listed in Rapoport and Guyer’s (1966) taxonomy of 2 x 2 games, and their associated properties of being Similarity Sensitive (SS) for the row and the column player. Payoffs are associated with their respective location in the following generic 2 by 2 matrix.

|  |  | Column Player | |
| --- | --- | --- | --- |
|  |  | A | B |
| Row player | A | AA row , AA col | AB row , AB col |
|  | B | BA row , BA col | BB row , BB col |

A game is similarity sensitive for the row player if: AA row > BB row and BA row > AB row, or if AA row < BB row and BA row < AB row. A game is similarity-sensitive for the column player if: AA col > BB col and BA col > AB col, or if AA col < BB col and BA col < AB col. Overall, the table shows 21 games that are similarity-sensitive for both row and column players, 36 games that are similarity-sensitive for only one of the players (either row or column), and 21 games that are non-similarity-sensitive for both players. Note that optimal switching points between the alternatives may be computed by comparing similarity perceptions of each player with the similarity thresholds of the game as prescribed by Subjective Expected Relative Similarity (SERS) theory.

|  |  |
| --- | --- |
|  |  |
| **Instructions for series recognition**  Thank you for agreeing to participate in this experiment.  In the present experiment you will be asked to recognize serial patterns created by different sources. For example, a coin tossed 4 times, may show ‘Heads’, then ‘Tails’, followed by ‘Heads’, and finally ‘Heads’ again. Another series can be created by a basketball player making or missing the basket.  Any series created by any source can be encoded. For example, if we toss a coin and code the outcomes ‘Heads’ as X and ‘Tails’ as O, the following series: Head, Head, Tail, Head will be coded as: X, X, O, X.  Similarly, coding baskets as X and misses as O, the sequence: Miss, Miss, Basket, Miss will be coded as: O, O, X, O.  In the experiment, you will see sequences collected from these two sources: coin tosses and basketball shots. For each of the presented series, you will be asked to use your judgment and intuition in order to decide which is the most likely source of the series.  All series are marked with the same symbols: @ or #, so the symbols themselves do not hint to the source of the sequence. The use of the symbols is of course consistent within each series.  Your task is to identify the source of each series in the experiment.  Good luck!  **Instructions for the PD training matrix**   \| The other player \| \| \|  \| \| --- \| --- \| --- \| --- \| \| Green \| Orange \|  \| You \| \| 10,20 \| 40, 40 \| Orange \| \| 30,30 \| 20, 10 \| Green \|   This is a table that provides an example of the game you will soon play.  Each participant in the game has two alternatives: to choose the green color or to choose the orange color. Your task is to choose one of the colors.  If you both chose the orange color, each of you will receive 40 points  If you both chose the green color, each of you will receive 30 points  If you choose the green color and the other participant chooses the orange color, you will receive 20 points and the other participant will receive 10 points.  If you choose the color orange and the other participant chooses the green color, you will receive 10 points and the second participant will receive 20 points.  The result of the game (i.e., your earnings) will be determined by the numbers listed in the cell representing the combined choices made by both participants.  The experiment will have only one round, which means that you will only have to make a single decision. Note that the outcome will be paid in real currency, hence you should carefully consider your choice.  Note the problem inherent in the game.  On the one hand, if your goal is to earn the largest amount, you will maybe choose the orange color, hoping that the other player will also choose the orange color, thus each of you will receive 40 points. However, the other player may choose the green color, and then your profit will be only 10 points, while the profit of the other player will be 20 points.  It seems that if the other player is about to choose the green color, it is better for you to also choose the green color. Then the result will be that each of you receives 30 points.  Of course it is possible the other player still prefers to choose the orange color, and then, if you chose the green color, you will obtain only 20 points. It seems that in this case it is better for you to choose the orange color and obtain 40 points.  But, of course the other player may choose the green color, in which case your profit will be 10 points ...  Now that you understand the complexity of the problem, you will get a new game with different payments. You need to think and carefully, consider all possible results, and choose one of the colors. As mentioned, the game will be played only once and its resulting outcome will be paid in real currency.  Note that you will make your decision alone without consulting the other participant.  Once you have made your choice, write it down on paper and seal it in the envelope.  Good luck!  **Comprehension test (administered as part of the instructions)**  If you choose the orange color, and the other player chooses the green color,  You will receive ___ points and the other player ___ points.  If you choose green, and the other player chooses orange,  You will receive ___ points and the other player ___ points.  If you both choose the orange color,  You will receive ___ points and the other player ___ points.  If you both choose the green color,  You will receive ___ points and the other player ___ points.  **The similarity measure**  Please estimate the degree to which thought processes of the other participant resemble your own way of thinking. Provide your answer by marking an X along the following continuum.    Not similar at all Maximally similar |  |
